# Using specific length amplified fragment sequencing to construct the high-density genetic map for *Vitis* (*Vitis vinifera* L. × *Vitis amurensis* Rupr.)

**DOI:** 10.3389/fpls.2015.00393

**Published:** 2015-06-04

**Authors:** Yinshan Guo, Guangli Shi, Zhendong Liu, Yuhui Zhao, Xiaoxu Yang, Junchi Zhu, Kun Li, Xiuwu Guo

**Affiliations:** College of Horticulture, Shenyang Agricultural UniversityShenyang, China

**Keywords:** grape, SLAF-seq, SNP, genetic map, linkage group

## Abstract

In this study, 149 F1 plants from the interspecific cross between ‘Red Globe’ (*Vitis vinifera* L.) and ‘Shuangyou’ (*Vitis amurensis Rupr*.) and the parent were used to construct a molecular genetic linkage map by using the specific length amplified fragment sequencing technique. DNA sequencing generated 41.282 Gb data consisting of 206,411,693 paired-end reads. The average sequencing depths were 68.35 for ‘Red Globe,’ 63.65 for ‘Shuangyou,’ and 8.01 for each progeny. In all, 115,629 high-quality specific length amplified fragments were detected, of which 42,279 were polymorphic. The genetic map was constructed using 7,199 of these polymorphic markers. These polymorphic markers were assigned to 19 linkage groups; the total length of the map was 1929.13 cm, with an average distance of 0.28 cm between each maker. To our knowledge, the genetic maps constructed in this study contain the largest number of molecular markers. These high-density genetic maps might form the basis for the fine quantitative trait loci mapping and molecular-assisted breeding of grape.

## Introduction

Grape (*Vitis vinifera*) is one of the most important fruit trees in the world and possesses high nutritional value. The demand for high-quality grape berries has been increasing in recent years. In 2013, 67 million tons grapes were produced globally over a cultivation area of 7 million hectare^1^. However, diseases and pest infestation as well as natural disasters adversely affect the grape growth, yield, and quality; therefore, modification of important quality characteristics and stress resistance has been a key target for grape breeders. Researchers use potential germplasms for crossing and composite-crossing to produce new cultivars with multiple favorable traits. However, the traditional cross-breeding techniques are time-consuming and require setting up many generations of hybridizations and back crosses. Recent advances in molecular biology have led to the construction of high-density molecular genetic maps and development of quantitative trait loci detection methods to identify target traits for grape breeding.

Lodhi et al. (1995) constructed the first molecular map of grapevine by using 422 random amplified polymorphic DNA (RAPD) markers, 16 restriction fragment length polymorphism (RFLP) markers and several isoenzyme markers and 60 F1 progenies of the cross between ‘Cayuga White’ × ‘Aurore.’ Since then, molecular genetic maps of grape have been constructed (Dalbó et al., 2000; Grando et al., 2003; Adam-Blondon et al., 2004; Riaz et al., 2004; Doligez et al., 2006; Lowe and Walker, 2006; Salmaso et al., 2008).

The previously published genetic maps for grape were mostly constructed using the F_1_ population as the mapping population; only some studies used F_2_ populations. Further, various types of molecular markers such as RAPD, amplified fragment length polymorphism (AFLP), single sequence repeat (SSR), sequence-related amplified polymorphism (SRAP), RFLP, and single nucleotide polymorphism (SNP) were used for the construction of the genetic maps. Of these markers, RAPD, AFLP, and SRAP markers lack repeatability and stability, and most of these markers have no sequence information; therefore, integrating information obtained using these markers with maps obtained using other markers is difficult. SSR, RFLP, and SNP markers are stable and reliable and can be used for map construction; however, SSR and RFLP marker development is a time-consuming and costly process, and only limited numbers of markers can be produced. Further, the efficiency of SSR markers is very low (Kennedy et al., 2003; West et al., 2006; Huang et al., 2009), and the uneven distribution of SSR markers in the genome hinders the construction of high-density maps. Numerous SNPs exist in the genome, and they are an ideal marker for constructing high-density genetic maps. However, detecting SNPs is difficult. Few linkage maps have been constructed using SNP markers, but the genome coverage degree of these maps was very low.

The next-generation sequencing technique can be used to detect large quantities of SNP markers in the entire genome. Several methods have been used for identifying SNPs such as restriction site associated DNA sequencing (Miller et al., 2007; Peterson et al., 2012) and genotyping-by-sequencing approach (Poland et al., 2012). By using restriction site associated DNA sequencing technique, high-density genetic maps for ryegrass (Pfender et al., 2011), barley (Chutimanitsakun et al., 2011), and grapevine (Wang et al., 2012) have been constructed; such maps have been constructed using the genotyping-by-sequencing approach for barley and wheat (Poland et al., 2012). Recently, Sun et al. (2013) developed a simplified genome sequencing technique [specific length amplified fragment sequencing (SLAF-seq)] that can efficiently explore large numbers of SNPs. The SLAF-seq technique has been used to construct high-density genetic maps for soybean (Qi et al., 2014) and sesame (Zhang et al., 2013).

In this study, we used the F_1_ population derived from an interspecific cross of ‘Red Globe’ (*V. vinifera* L.) × ‘Shuangyou’ (*Vitis amurensis* Rupr.) and the parent populations to construct a high-density genetic map of grape (*V. amurensis*). This map consists of numerous SNP and insertion-deletion (In-Del) markers (7,199 markers). It can be used for the fine quantitative trait loci mapping of important traits in grape, such as the resistance of cold and disease.

## Materials and Methods

### Plant Material and DNA Extraction

In the autumn of 2009, seeds were collected from the F_1_ population of a cross of ‘Red Globe’ (*V. vinifera* L.) and ‘Shuangyou’ (*V. amurensis* Rupr.). Between October 2009 and February 2010, the stratification treatment was performed. The hybrid seeds were sown in a greenhouse in March 2010. Young plants were transferred to an experimental field at the Shenyang Agriculture University in Shenyang (126°33′41^′′^E, 41°49′24^′′^N), Liaoning Province, China, and planted from April to June in batches. In all, 777 individuals were obtained, of which 149 individuals and their parents were used as the mapping population. Young healthy leaves from the two parents and progenies were collected, and genomic DNA was extracted using the CTAB method (Hanania et al., 2004). DNA was quantified using an ND-1000 spectrophotometer (NanoDrop, Wilmington, DE, USA) and observed using electrophoresis on 0.8% agarose gels with lambda DNA as a standard.

### Genotyping

The SLAF-seq method was used to genotype the 149 progeny individuals and the two parents, as described previously (Sun et al., 2013) with slight modifications. The genomic DNA from each sample was treated with *Rsa*I*, Hae*III (NEB, Ipswich, MA, USA), T4 DNA ligase (NEB), and ATP (NEB), and maintained at 37°C. The restriction-ligation reaction solutions were diluted and mixed with dNTP, Taq DNA polymerase (NEB), and *Hae*III primer for polymerase chain reaction (PCR) analyses. The PCR products were purified using E.Z.N.A. Cycle Pure Kit (Omega, London, UK). The purified PCR products were pooled and incubated at 37°C with *Hae*III, T4 DNA ligase, ATP, and Solexa adapter. After incubation, the products were purified using Quick Spin column (Qiagen, Venlo, Netherlands) and electrophoresed on 2% agarose gel. Gel Extraction Kits (Qiagen) were used to isolate the SLAF products that ranged from 550 to 600 bp (including the adapter sequence indexes and adaptors). The products were then processed for a second PCR by using the Phusion Master Mix (NEB) and Solexa Amplification primer mix. According to the Illumina sample preparation guide (Illumina, Inc., San Diego, CA, USA), the PCR products were gel purified, and SLAFs of 314–414 bp were selected for paired-end sequencing on an Illumina HiSeq 2500 sequencing platform performed by the Beijing Biomarker Technologies Corporation^2^. DNA sequence reads were 200 bp in size.

According to the barcode sequences, raw reads were demultiplexed to individuals. Subsequently, low-quality reads (quality score < 30) were filtered out. After the barcodes were trimmed from the reads, reads of 100 bases from the same samples were mapped onto the grape genome sequence by using SOAP denovo2 software (Luo et al., 2012). SOAP2 was used with the default parameters, but *r* = 0, *M* = 4, *m* = 50, and *x* = 1,000, where *r* = 0 indicates that multiple matches are not reported, *M* = 4 detects the best hits, and *m* = 50 with *x* = 1,000 suggests that the insert size is 50–1,000 bp. Sequences mapped to the same position were defined as one SLAF loci. In each SLAF, most polymorphic loci found between the parents were SNPs. All polymorphic SLAF loci were genotyped with consistency in the offspring and parental SNP loci.

All SLAF markers were filtered four times, and the quality was assessed as described by Sun et al. (2013). The markers with less than 3 SNPs and average sequencing depth higher than 3 were treated as high-quality SLAF markers. These markers were used to construct high-density genetic maps.

### Linkage Map Construction

Since next-generation sequencing data might include many genotyping errors and deletions, which could reduce the quality of the high-density linkage maps, High Map Strategy was used to order the SLAF markers and correct the genotyping errors in the linkage groups(LGs; Liu et al., 2014a). All high-quality SLAF markers were allocated to 19 LGs on the basis of their locations on chromosomes. Detaily MSTmap algorithm was used to order the SLAF markers (Wu et al., 2008), and the SMOOTH algorithm (Van et al., 2005) was used to correct the genotyping errors as per the marker ordering. All LGs were processed as follows: a primary marker was used to order the LGs by their location on chromosomes; according to the relationship between the ordered markers, genotyping errors or deletion were corrected using SMOOTH algorithm; the minimum spanning tree map was used to order the map; and SMOOTH algorithm was used to correct the newly ordered genotypes. After four or more cycles of this processing, 19 high-quality maps were obtained. The Kosambi mapping function was used to estimate the map distances (Kosambi, 1944).

## Results

### Analysis of SLAF-seq Data and SLAF Markers

**FIGURE 1:**
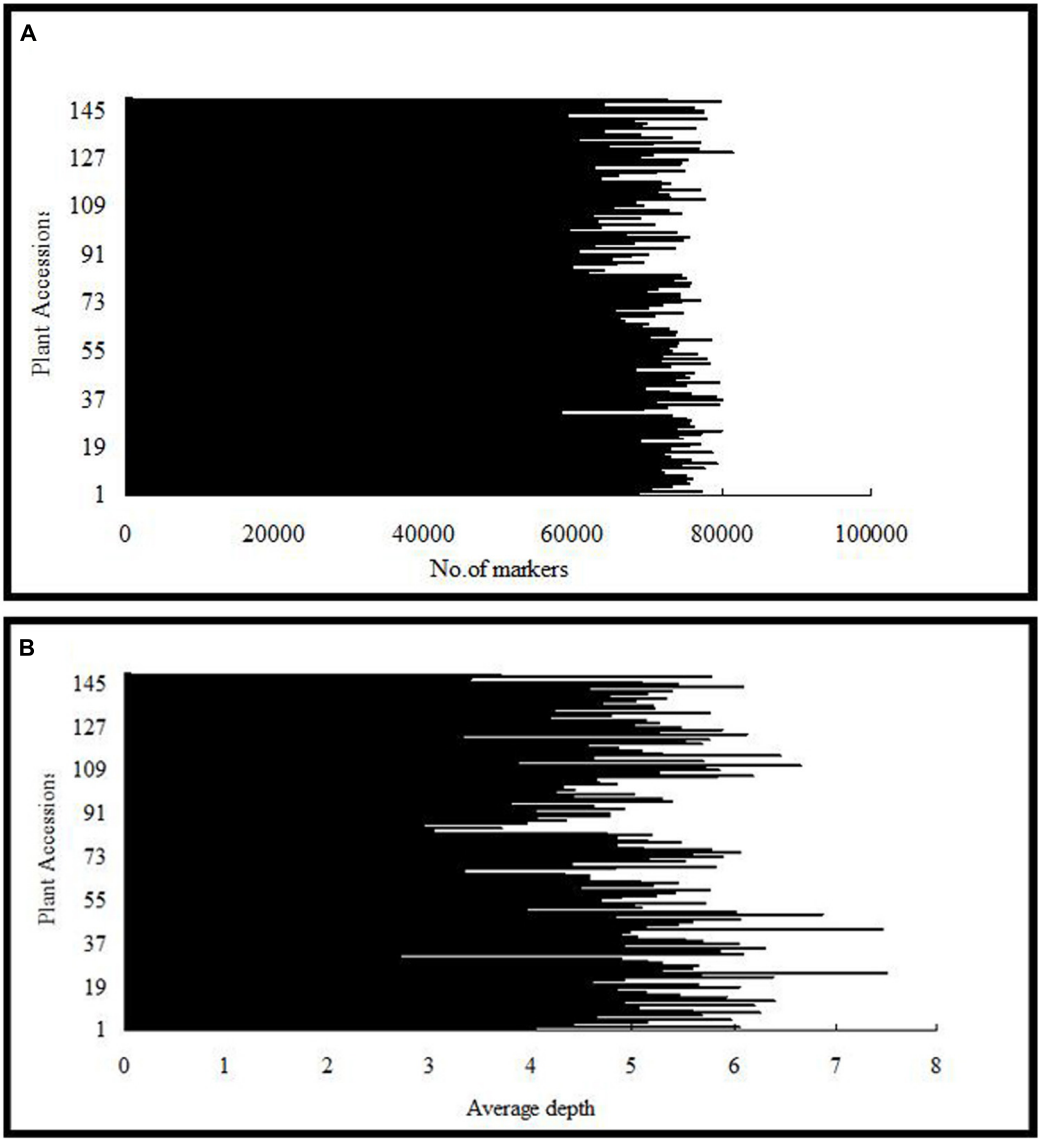
**Number of markers **(A)** and average sequencing depth **(B)** expressed as genome equivalents of the 149 individuals of F_1_ population represented in the X-axis**.

After the preprocessing, 41.282 Gb raw data were obtained, which consisted of 206,411,693 paired-end reads of ∼100 bp in length. Of these, 80.60% bases were of high quality, with quality scores of at least 30 (Q30, indicating a 0.1% chance of an error, and thus 99.9% confidence). The guanine–cytosine content was 37.83%. Subsequently, 115,629 SLAFs were detected, and their average sequencing depth was 41.79 for ‘Red Globe’; 40.74 for ‘Shuangyou’; and 5.18 for each progeny (**Figure 1**).

**FIGURE 2:**
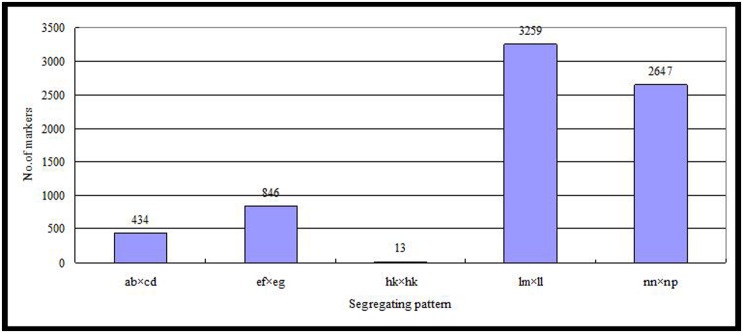
**Number of markers in each of the five segregation patterns**.

Of the 115,629 SLAFs, 42,279 were polymorphic, with a polymorphism rate of 36.56%. The number of polymorphic SLAF markers per chromosome ranged from 1,426 in chromosome 3 to 3,005 in chromosome 18 (**Table 1**). The genotype coding for the polymorphic SLAF tags was conducted on the basis of the genotype of the parents. In all, 27,985 tags were coded, of which, 13,738 tags could be used to construct the genetic maps, with an effective polymorphism rate of 11.88%. After filtering out the low quality SLAFs lacking parent information and the depth less than 10X, 7,199 markers were obtained that could be used to construct a genetic map by using the criteria of segregation distortion (*P* < 0.05); these markers were classified into five segregation patterns (**Figure 2**). The average sequencing depths of these 7,199 markers were 68.35-fold for ‘Red Globe,’ 63.65-fold for ‘Shuangyou,’ and 8.01-fold for each individual progeny.

**Table 1:** The description of basic characteristics for the 19 linkage groups.

Linkage groups (LG)	Total marker			Total distance (cm)			SLAFs (consensus map)		
	Red Globe	Shuangyou	Consensus map	Red Globe	Shuangyou	Consensus map	SLAF Numner	Polymorphic SLAF
LG01	293	296	462	105.45	110.24	114.01	6651	2405
LG02	208	141	328	70.06	55.63	67.07	5779	2183
LG03	42	200	326	16.36	68.39	66.88	4858	1426
LG04	214	269	450	79.92	84.69	86.04	7397	2807
LG05	369	327	548	214.47	133.98	177.31	6904	2627
LG06	340	319	503	105.85	101.87	103.86	5744	2328
LG07	305	264	455	136.13	125.40	131.78	5653	2257
LG08	279	192	431	105.13	76.30	95.81	6285	2504
LG09	125	153	257	57.58	64.44	64.43	5208	1831
LG10	152	115	244	48.49	58.28	57.57	4775	1831
LG11	234	265	401	106.48	105.33	106.92	6286	2276
LG12	115	218	311	114.55	85.47	101.22	6070	2109
LG13	64	260	319	21.82	67.49	67.47	6334	2289
LG14	237	376	504	122.41	143.88	140.33	8190	2849
LG15	59	242	246	174.22	103.74	138.98	4918	1447
LG16	162	197	285	110.92	94.89	103.25	4931	1729
LG17	232	232	370	90.84	81.90	86.37	5496	2045
LG18	262	227	460	133.02	87.65	111.44	8305	3005
LG19	248	269	389	118.21	97.23	108.39	5845	2291
Total	3940	4552	7199	1931.91	1746.80	1929.13	115629	42279

### Basic Characteristics of the Genetic Map

In all, 7,199 genomic regions were covered. The LGs were numbered according to the chromosome numbers. The length of the genetic map was 1929.13 cm, and the average distance between two adjacent markers was 0.28 cm (**Table 2**, Supplementary Table S1). The average length of the 19 LGs was 101.11 cm, and the average number of markers for each LG was 397. LG10 was the shortest group with a length of 57.57 cm, and the LG5 was the longest group with the length of 177.31 cm (**Table 1**). LG4 was the highest density group, containing 450 markers, and the average marker density was 0.19 cm. LG5 was the longest group and contained 548 markers with an average genetic distance of 0.57 cm. LG10 was the shortest group and contained 244 markers with an average genetic distance of 0.24 cm. In these 19 LGs, the average percentage of markers having a gap distance of ≤5 cm was 98.62%, but LG8 and LG16 had no gap distance of ≥5 cm, and the largest gap was 15.14 cm, which was located on LG5 (**Table 2**, Supplementary Table S1).

**Table 2:** The description of basic characteristics for the 19 LGs.

Linkage groups (LG)	Average distance (cm)			Gaps ≤ 5 (max gap)		
	Red Globe	Shuangyou	Consensus map	Red Globe	Shuangyou	Consensus map
LG01	0.36	0.39	0.25	99.32% (12.27)	98.60% (6.41)	99.35% (4.54)
LG02	0.34	0.40	0.21	98.55% (12.09)	98.57% (5.68)	99.69% (3.47)
LG03	0.40	0.34	0.28	92.68% (2.76)	97.99% (7.21)	99.15% (7.21)
LG04	0.38	0.32	0.19	96.71% (7.99)	98.88% (4.93)	98.22% (3.20)
LG05	0.58	0.41	0.32	97.55%(29.41)	98.47% (7.59)	98.90% (15.14)
LG06	0.31	0.32	0.21	98.82% (9.59)	98.74% (6.44)	99.00% (5.19)
LG07	0.45	0.48	0.29	97.70% (9.12)	95.82% (11.07)	99.56% (5.54)
LG08	0.38	0.40	0.22	99.28% (7.99)	98.43% (4.20)	100.00% (2.44)
LG09	0.46	0.42	0.25	91.94% (10.41)	94.08% (9.59)	94.53% (4.58)
LG10	0.32	0.51	0.24	100.00% (3.21)	97.37% (9.05)	99.59% (8.37)
LG11	0.46	0.40	0.27	97.00% (12.75)	99.24% (5.68)	99.75% (3.63)
LG12	1.00	0.39	0.33	91.23% (53.61)	98.62% (7.21)	96.77% (7.24)
LG13	0.35	0.26	0.21	74.60% (2.76)	98.84% (4.93)	99.06% (2.86)
LG14	0.52	0.38	0.28	97.03% (30.04)	95.73% (7.40)	98.81% (8.86)
LG15	3.00	0.43	0.57	77.59% (10.05)	98.76% (7.68)	99.18% (7.38)
LG16	0.69	0.48	0.36	98.76% (43.04)	98.98% (4.20)	100.00% (4.25)
LG17	0.39	0.35	0.23	91.34% (19.91)	96.54% (3.47)	93.22% (10.25)
LG18	0.51	0.39	0.24	96.93% (21.46)	99.56% (4.20)	99.56% (11.00)
LG19	0.48	0.36	0.28	98.38% (17.50)	99.25% (8.78)	99.48% (5.59)
Average	0.60	0.39	0.28	94.50%	98.02%	98.62%

‘Gaps ≤ 5’ represents the percentage of gaps in which the distance between two adjacent markers was smaller than 5 cm.

There were 3,940 markers in the female map with a full length of 1931.91 cm. For the female population, the genetic length of LGs ranged from 16.36 cm (LG10) to 214.47 cm (LG5). LG6 was the highest density group, which contained 340 markers, and the average marker density was 0.31 cm. LG15 was the lowest density group, which contained 59 markers, and the average marker density was 3.00 cm. LG5 was the largest LG and contained 369 markers, covering a length of 214.47 cm with an average inter-marker distance of 0.58 cm. LG3 was the shortest group and contained 42 markers with the length of 16.36 cm and an average genetic distance of 0.40 cm (**Table 1**, Supplementary Figure S1).

There were 4,552 markers in the male map with a length of 1746.8 cm. LG2 was the shortest group with the length of 55.63 cm and contained 141 molecular markers. The average genetic distance was 0.40 cm. LG14 was the longest group with the length of 143.88 cm and contained 376 molecular markers; the average genetic distance was 0.38 cm. LG13 was the highest density group, which contained 260 markers, and the average marker density was 0.26 cm. LG10 was the lowest density group, which contained 115 markers, and the average marker density was 0.51 cm (**Table 1**, Supplementary Figure S2).

### The Distribution of the Three Markers Types on the Genetic Map

The genetic maps constructed using SLAF sequencing contained three types of molecular markers: 7124 ‘SNP_only’-type, 7 ‘Indel_only’-type, and 68 ‘SNP&Indel’ markers. The ‘SNP_only’ was the major marker type and occupied 98.96% of the markers. ‘Indel_only’ markers were present only in LG1, LG6, LG7, and LG19; two ‘Indel_only’ markers each were present in LG1, LG6, and LG7 and only one existed in LG19. ‘SNP & InDel’ markers existed in all the 19 LGs. The number of ‘SNP & InDel’ markers in each LG ranged from 1 to 9, and only LG1 had 9 ‘SNP & InDel’ markers.

### Visualization and Evaluation of the Genetic Map

The quality of the genetic map was evaluated using haplotype maps and heat map. Haplotype map can be used to detect double crossover populations, suggesting genotyping errors. It can also show the reorganization of each individual. The 7,199 SLAF markers were used to construct haplotype maps for each of the 149 progenies and parental controls, as described by West et al. (2006). Haplotype maps directly reflected the recombination events of each individual. Most of the recombination blocks could be easily identified in the haplotype maps. These maps suggested that the double crossover and missing ratio on the genetic maps were low (<1.5% had heterozygous fragments and <0.6% were missing). All LGs were distributed uniformly. Therefore, the population used in this study was suitable for the construction of genetic maps and performing genetic analysis.

In each LG, the relationship of recombination between all the markers was reflected by the heat map; this was subsequently used to detect ordering errors. This was also used to evaluate the quality of genetic maps by using pair-wise recombination values for the 7,199 SLAF markers. Most of the LGs had good visualization in general.

## Discussion

### The Feasibility and Advantages of SLAF Sequencing in Marker Development

The key step for high-density map construction is to develop numerous stable and reliable molecular markers. In this study, SNP and InDel markers were used to construct a high-density genetic map for grape.

Specific length amplified fragment sequencing is a high throughput sequencing technique based on bioinformatics. It can be used for large-scale genotyping, which plays an important role in genetic linkage analysis. Unlike traditional molecular marker sequencing techniques, SLAF sequencing can provide high marker density and good uniformity. It also has many advantages (Sun et al., 2013).

Thus far, several high-density genetic maps have been constructed using SLAF-seq. Zhang et al. (2013) developed the first high-density genetic map in sesame; Qi et al. (2014), in soybean; and Xu et al. (2015) in cucumber. Chen et al. (2013) and Huang et al. (2013) conducted such genetic analysis in kiwi fruit and wheat, and the marker number produced by SLAF-seq was more than 1,000, which was considerably higher than the numbers of markers used in conventional maps.

In this study, the total quantity of SLAF-seq data generated from the parents and progenies was 41.282 Gb, including 115,629 high-quality SLAF tags, of which 42,279 were polymorphic. The final high-density genetic map contained 7,199 molecular markers. SLAF-seq has been able to detect numerous high-quality markers, especially in species with low polymorphism (Xie et al., 2010). In this study, the percentage of valid polymorphic markers between two parents was 11.88%. This percentage was not very high, but all the chromosomes were covered by polymorphic tags, whose number ranged from 1,426 to 3,005 on each chromosome. Therefore, SLAF-seq technology can be considered as a cost-effective technique to successfully develop chromosome-specific molecular markers for fruit trees, with high specificity and stability.

### Evaluation and Usage of Genetic Maps

In this study, SLAF-seq technique was used to construct a molecular genetic map for grape fruit; this map contained the largest number of molecular markers compared to the published genetic maps for grape. The quality of the genetic map was evaluated by creating a single source figure and heat map, or by comparing the colinearity between the genetic map and genome. The origin of the major segment in each chromosome was consistent. Further, the ratio of double recombination for markers and marker deficiency pattern were 1.5 and 0.6%, respectively, indicating that the marker order was accurate in each LG. The heat map of the marker exchange relationship was used for evaluating the linkage relationship among the markers. The trend of recombination change between the markers was consistent with the marker order in each LG. The colinearity between the genetic map and genome was higher, indicating the high quality of the map. The order of most markers was consistent with those in the reference genome, indicating a good colinearity. Thus, the result of recombination rate was accurate.

In this study, grape (*V. vinifera* L. cv. Red Globe) was used as the maternal parent. It is the main cultivar and accounts for over 20% of the total grape area in China. It has a good berry quality, large cluster size, large berry size, low acid content, late-ripening, firm flesh, and good endurance of storage and transportation, whereas it has poor cold and pathogen resistance such as elsinoe anthracnose, downy mildew and so on (Liu et al., 2014b). The paternal parent used was ‘Shuangyou’ (*V. amurensis* Rupr.), which is a bisexual cultivar native to China; this cultivar is extensively cultivated in northeast China. It has high cold resistance and can withstand a temperature of -40°C. It also has high disease resistance such as elsinoe anthracnose, white rot, powdery mildew, and so on (Liu et al., 2014b). However, this plant bears small berries, has high content of acid, and is an early ripening variety. In China, it is used for making *amurensis* wine and grape juice. For over several years, we have been collecting the phenotypic data, including sugar content, acid content, cluster weight, berry weight, cold hardiness, and pathogen resistance, of ‘Red Globe,’ ‘Shuangyou,’ and their progenies. Remarkable segregation was found in these characters. In the future, we intend to map the quantitative trait loci for these traits on the genetic map; the markers associated with the agronomic traits and cold and disease resistance will also be determined. This information might be used for maker-assisted breeding and gene cloning based on the map developed in the study. Considering the distant hybridization between the parents, there might be a possibility of obtaining hybrid offspring that have the favorable traits of both the parents.

## Conflict of Interest Statement

The authors declare that the research was conducted in the absence of any commercial or financial relationships that could be construed as a potential conflict of interest.

## Footnotes

1 http://faostat3.fao.org/browse/Q/QC/E

2 http://www.biomarker.com.cn

## Supplementary material

**Table S1 | Information of the marker on the19 LGs.**

**Figure S1 | Grape high density genetic map of the female parent.** The map was constructed based on SLAF markers.

**Figure S2 | Grape high density genetic map of the male parent.** The tructed based on SLAF markers.
