# Supplementary figures and images for: Using specific length amplified fragment sequencing to construct the high-density genetic map for Vitis (Vitis vinifera L. × Vitis amurensis Rupr.)
Source: Front Plant Sci. 2015 Jun 4;6:393. doi: 10.3389/fpls.2015.00393 (PMC4454882; doi:10.3389/fpls.2015.00393)

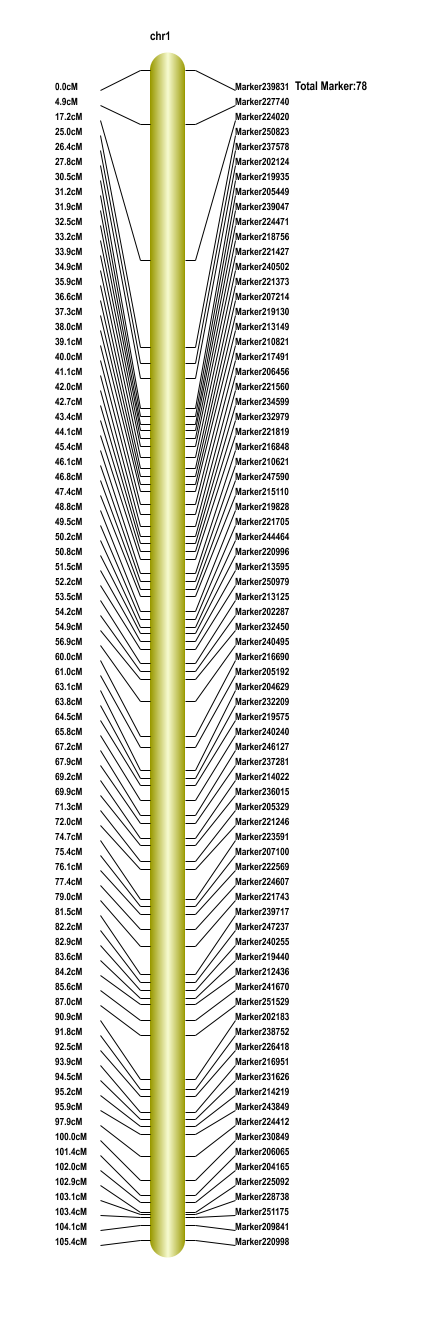

Supplement: Supplementary file 2 [file Presentation_2.ZIP › female map/Grape.chr1.png]

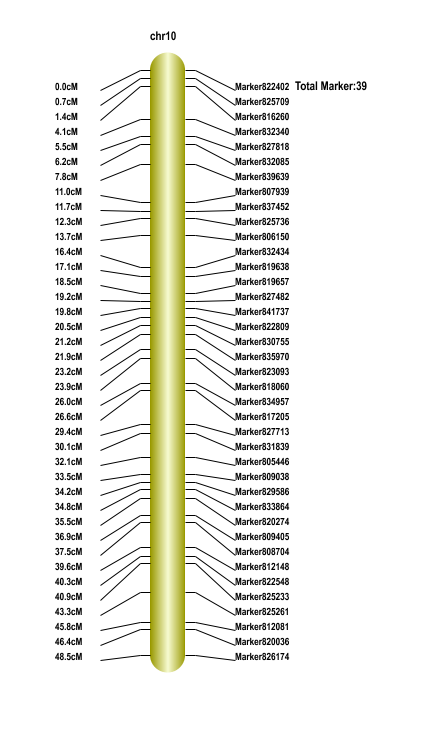

Supplement: Supplementary file 2 [file Presentation_2.ZIP › female map/Grape.chr10.png]

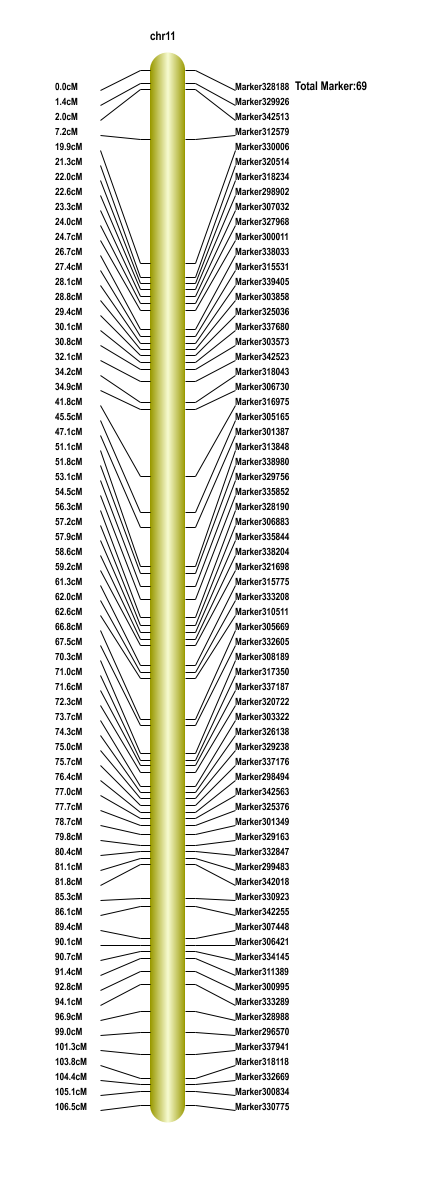

Supplement: Supplementary file 2 [file Presentation_2.ZIP › female map/Grape.chr11.png]

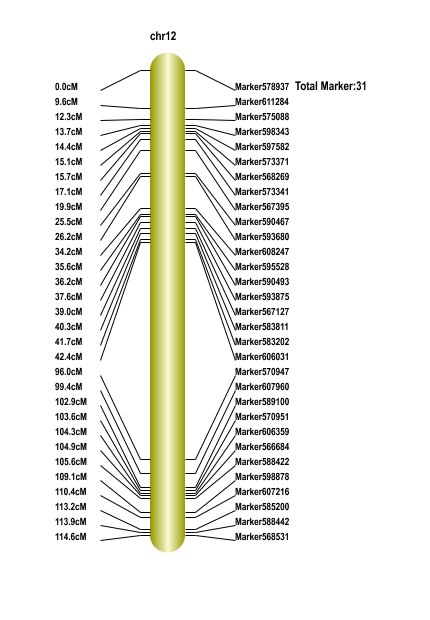

Supplement: Supplementary file 2 [file Presentation_2.ZIP › female map/Grape.chr12.png]

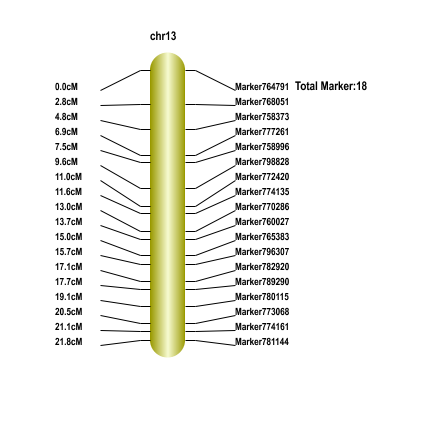

Supplement: Supplementary file 2 [file Presentation_2.ZIP › female map/Grape.chr13.png]

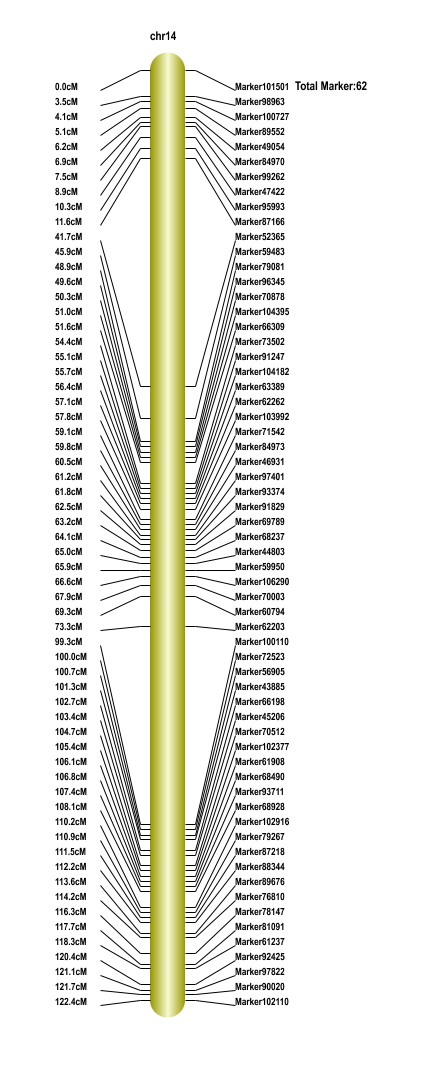

Supplement: Supplementary file 2 [file Presentation_2.ZIP › female map/Grape.chr14.png]

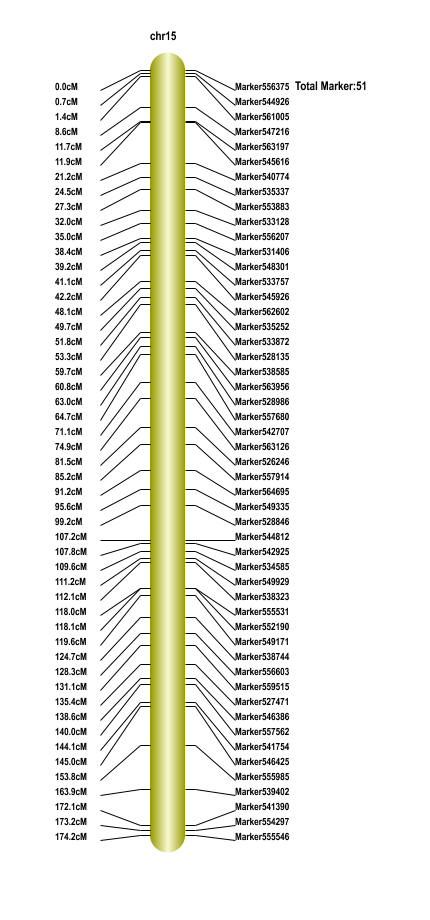

Supplement: Supplementary file 2 [file Presentation_2.ZIP › female map/Grape.chr15.png]

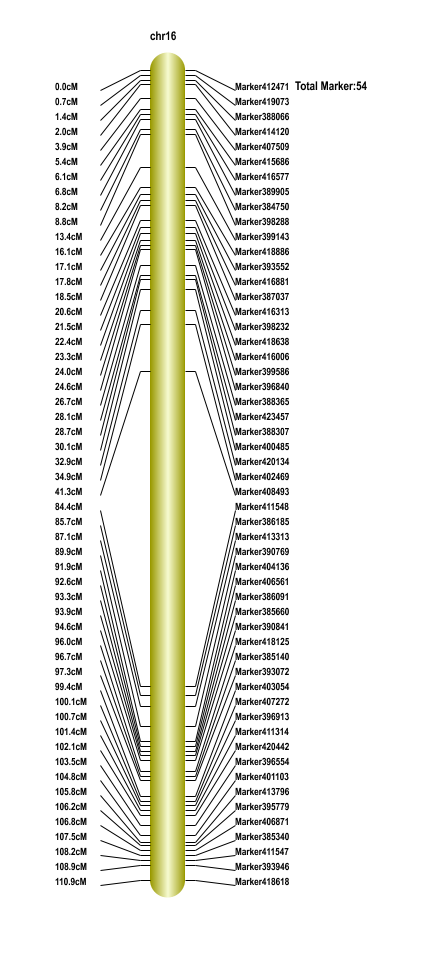

Supplement: Supplementary file 2 [file Presentation_2.ZIP › female map/Grape.chr16.png]

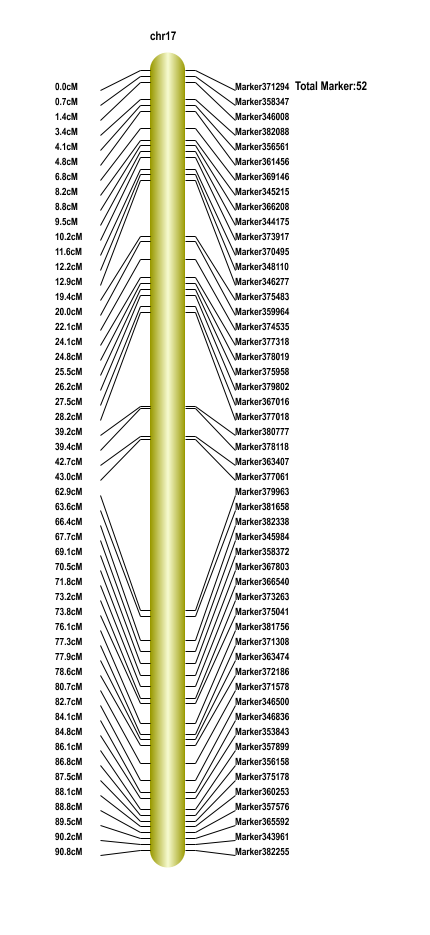

Supplement: Supplementary file 2 [file Presentation_2.ZIP › female map/Grape.chr17.png]

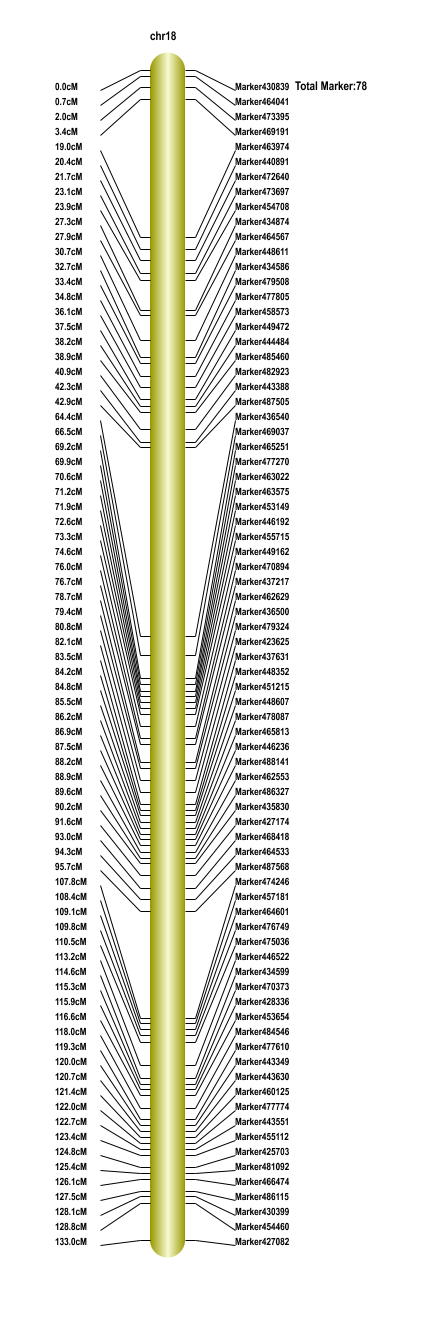

Supplement: Supplementary file 2 [file Presentation_2.ZIP › female map/Grape.chr18.png]

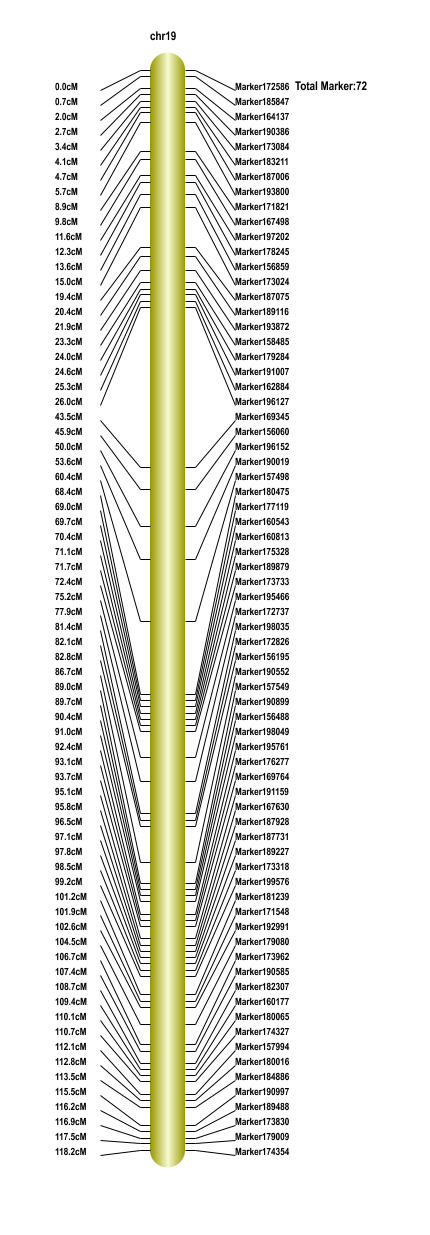

Supplement: Supplementary file 2 [file Presentation_2.ZIP › female map/Grape.chr19.png]

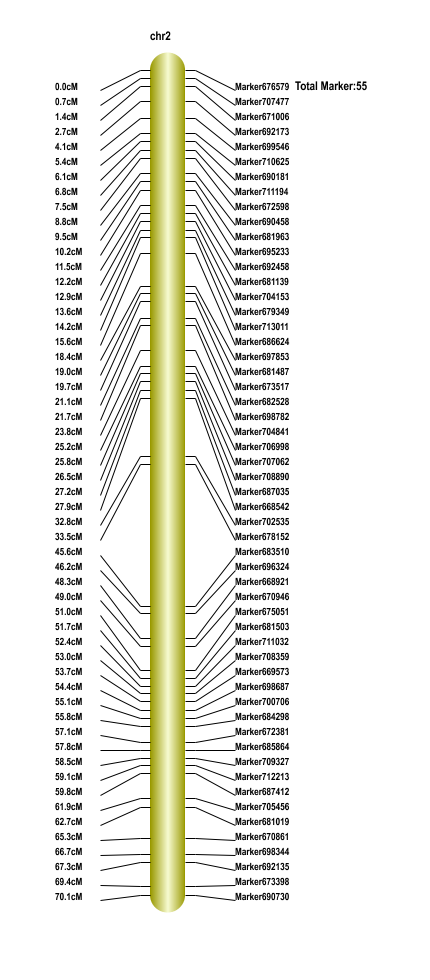

Supplement: Supplementary file 2 [file Presentation_2.ZIP › female map/Grape.chr2.png]

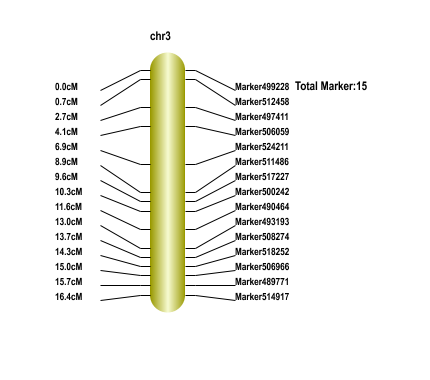

Supplement: Supplementary file 2 [file Presentation_2.ZIP › female map/Grape.chr3.png]

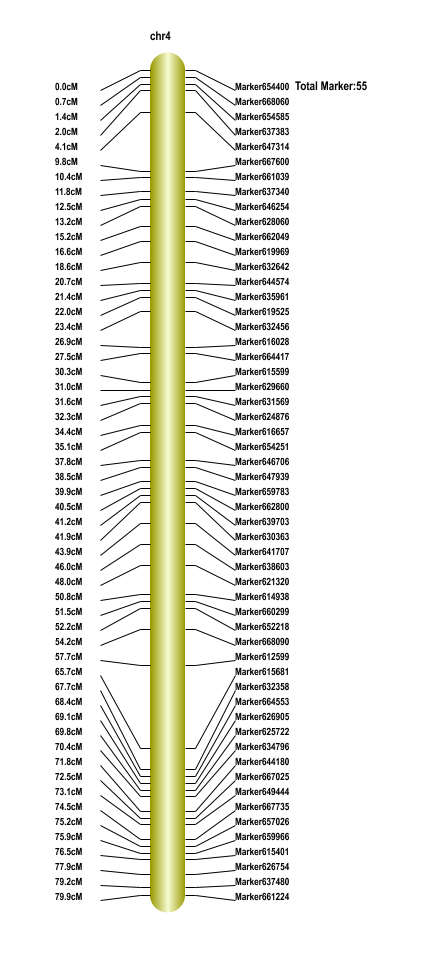

Supplement: Supplementary file 2 [file Presentation_2.ZIP › female map/Grape.chr4.png]

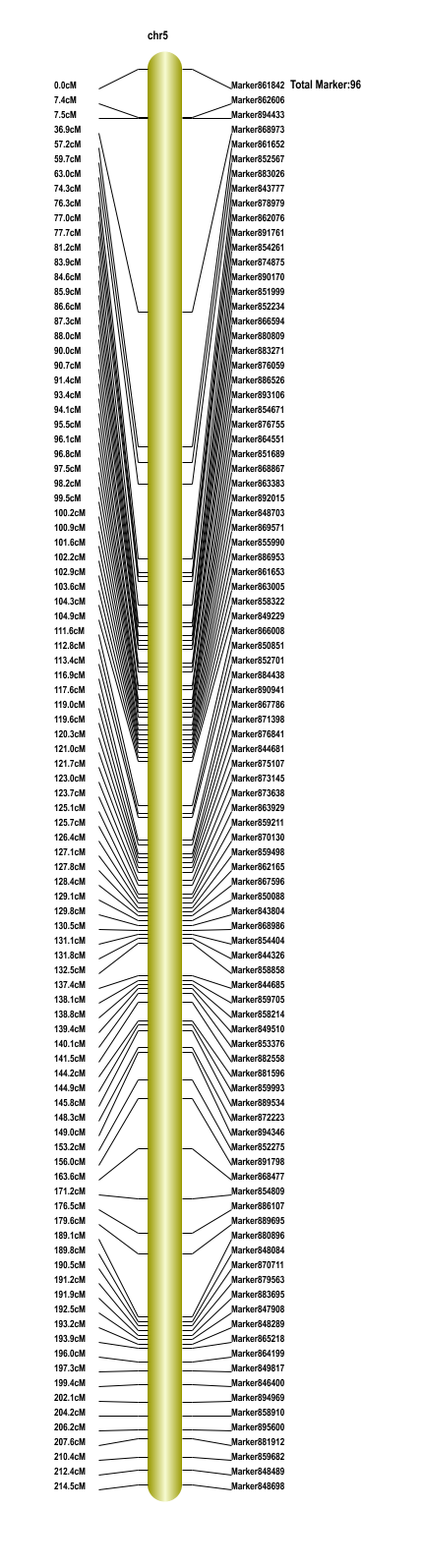

Supplement: Supplementary file 2 [file Presentation_2.ZIP › female map/Grape.chr5.png]

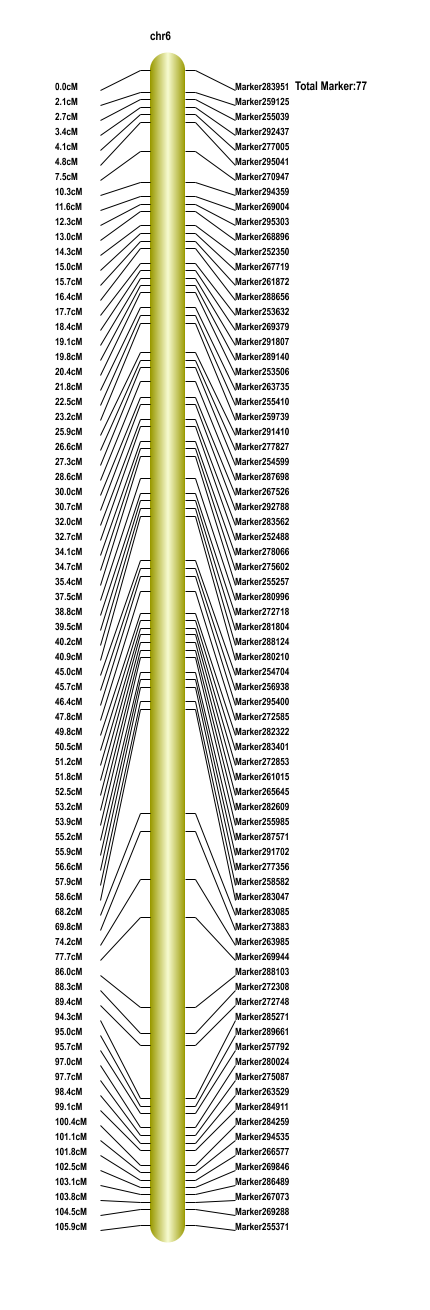

Supplement: Supplementary file 2 [file Presentation_2.ZIP › female map/Grape.chr6.png]

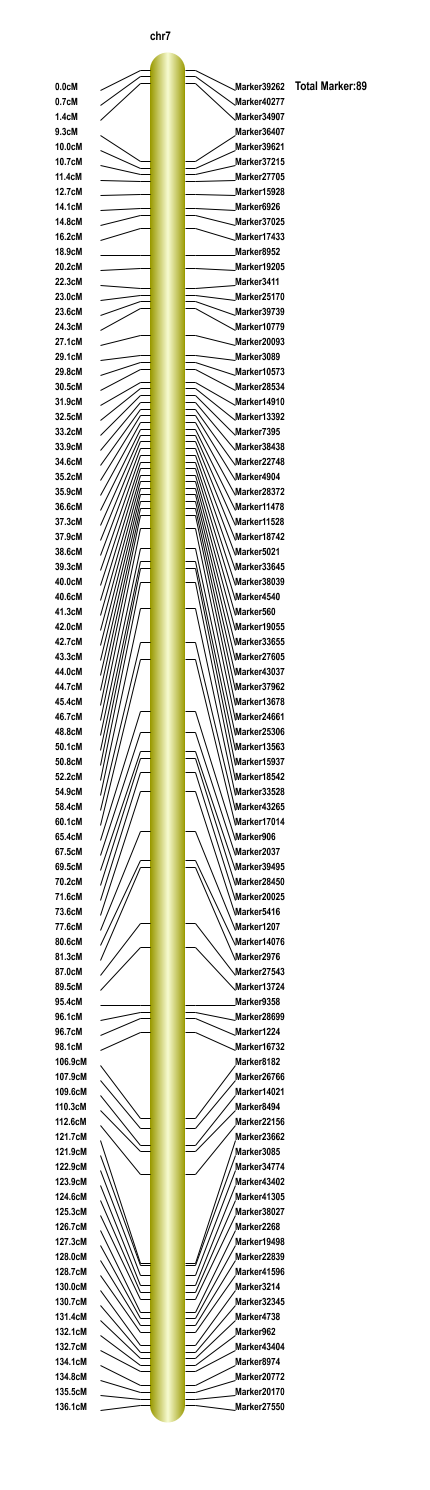

Supplement: Supplementary file 2 [file Presentation_2.ZIP › female map/Grape.chr7.png]

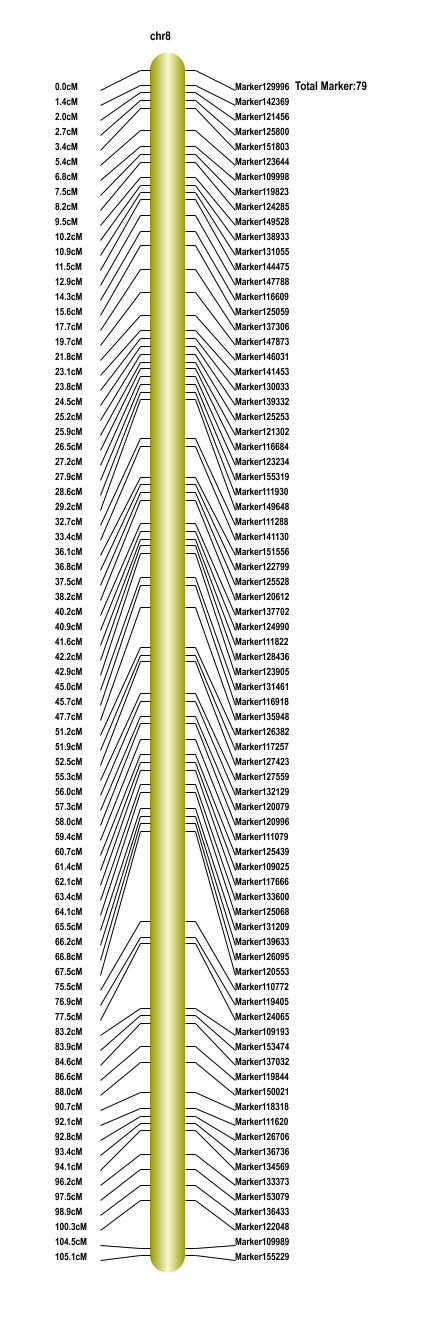

Supplement: Supplementary file 2 [file Presentation_2.ZIP › female map/Grape.chr8.png]

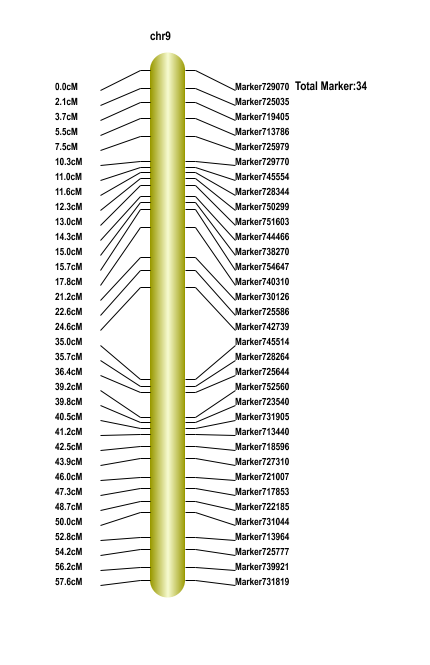

Supplement: Supplementary file 2 [file Presentation_2.ZIP › female map/Grape.chr9.png]

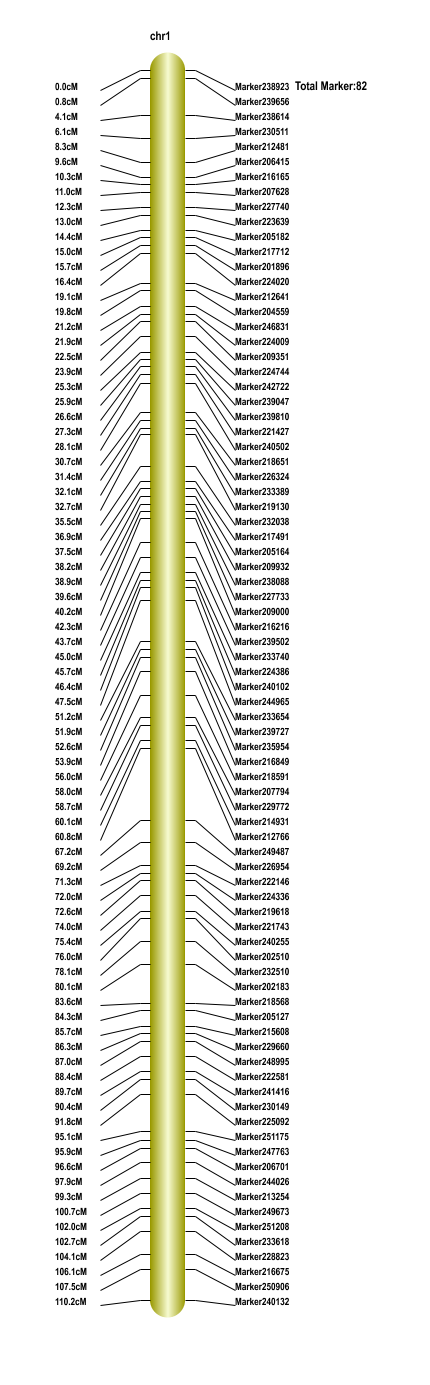

Supplement: Supplementary file 3 [file Presentation_3.ZIP › male map/Grape.chr1.png]

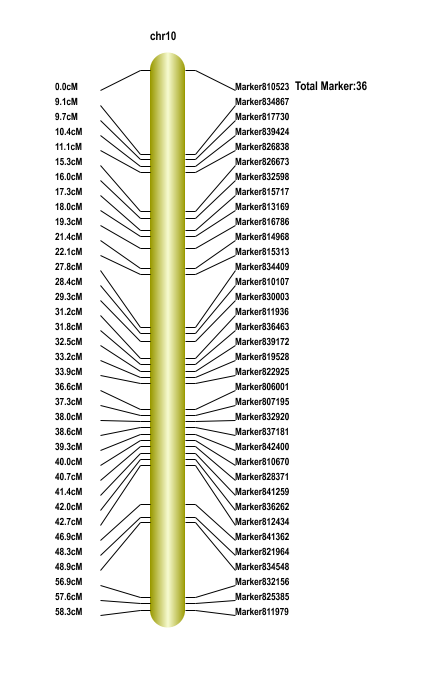

Supplement: Supplementary file 3 [file Presentation_3.ZIP › male map/Grape.chr10.png]

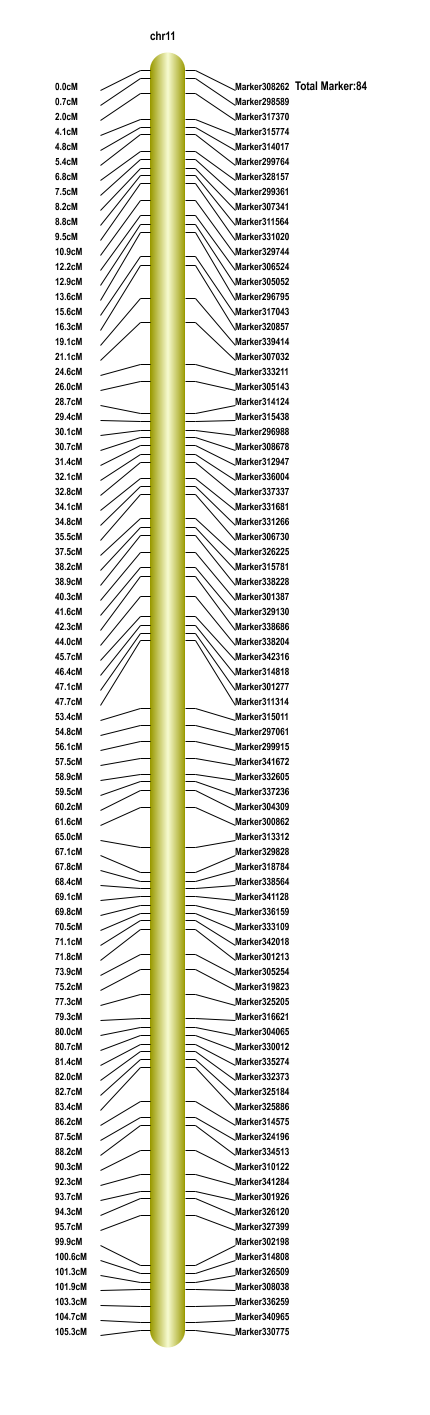

Supplement: Supplementary file 3 [file Presentation_3.ZIP › male map/Grape.chr11.png]

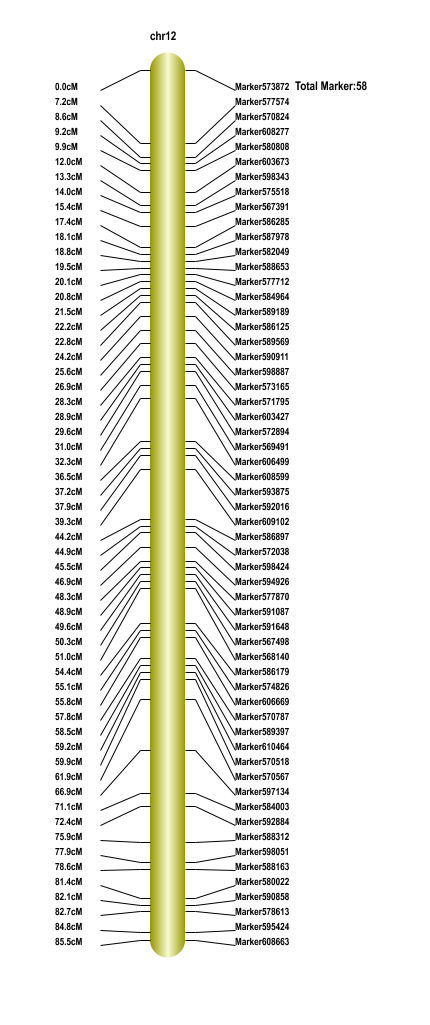

Supplement: Supplementary file 3 [file Presentation_3.ZIP › male map/Grape.chr12.png]

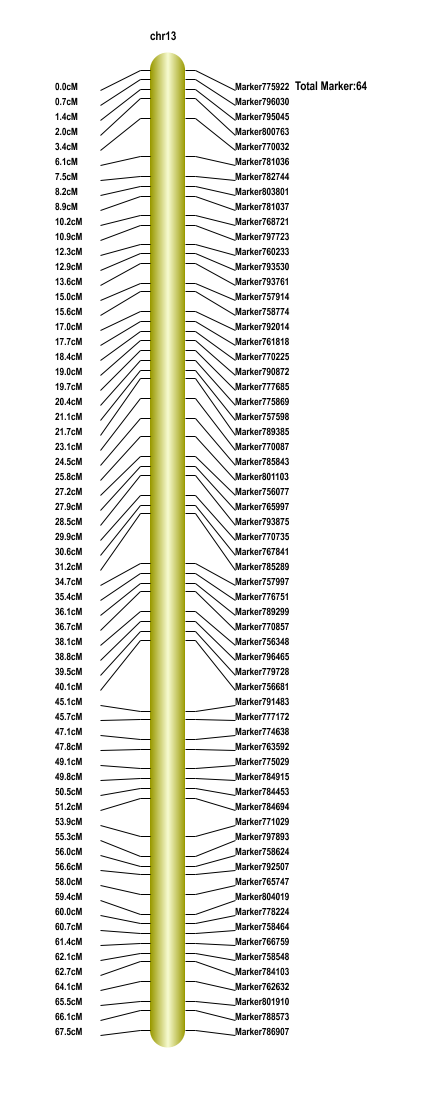

Supplement: Supplementary file 3 [file Presentation_3.ZIP › male map/Grape.chr13.png]

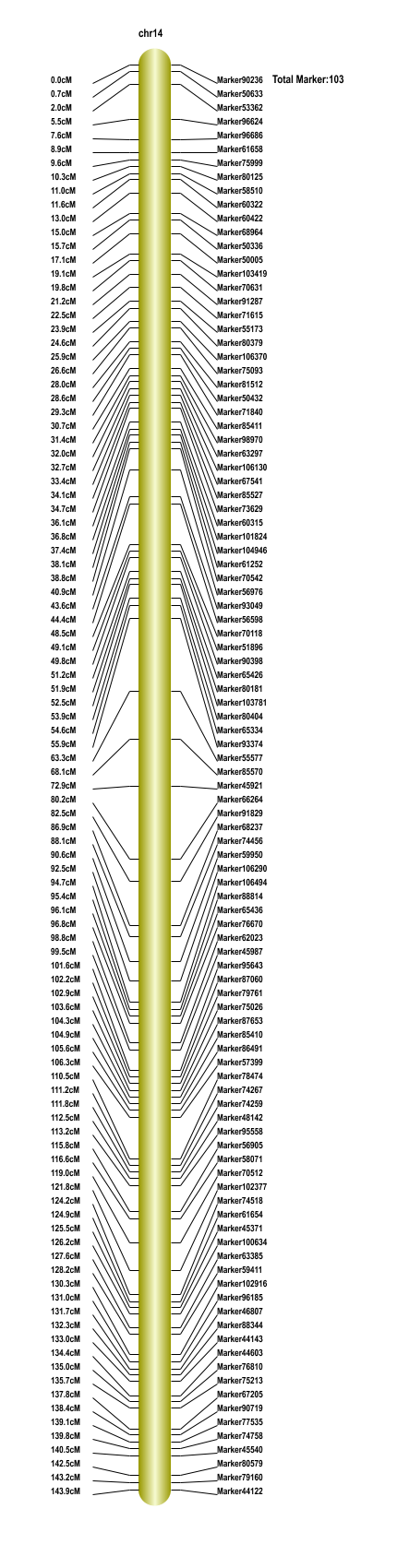

Supplement: Supplementary file 3 [file Presentation_3.ZIP › male map/Grape.chr14.png]

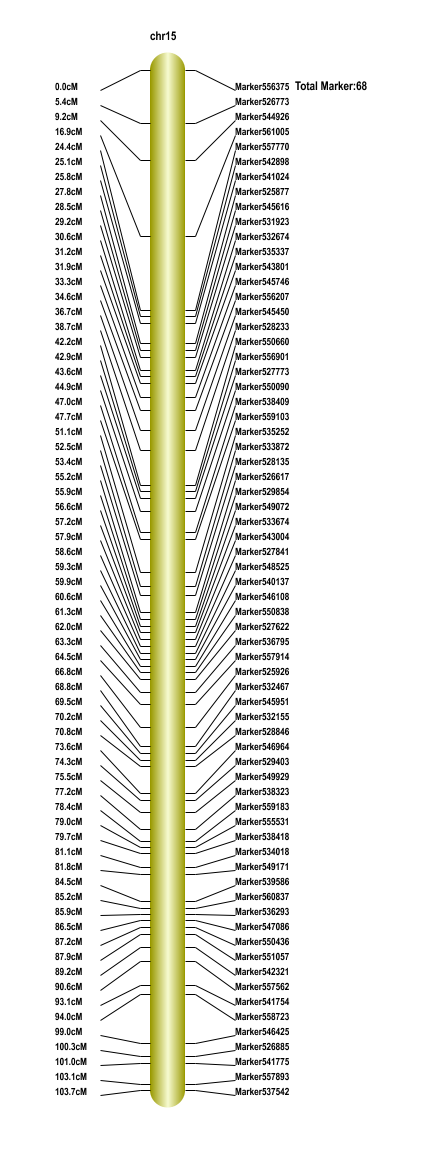

Supplement: Supplementary file 3 [file Presentation_3.ZIP › male map/Grape.chr15.png]

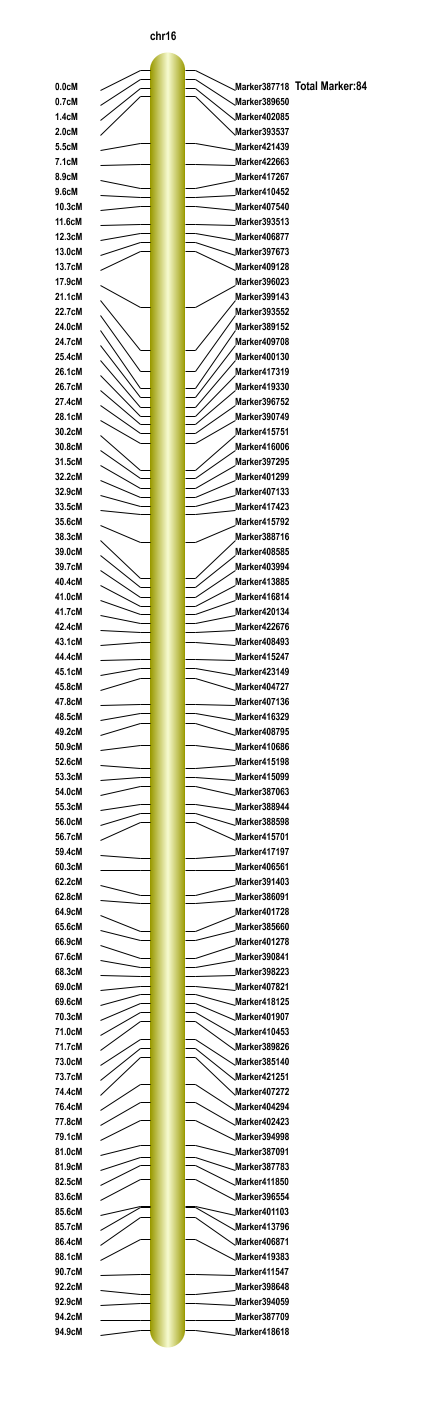

Supplement: Supplementary file 3 [file Presentation_3.ZIP › male map/Grape.chr16.png]

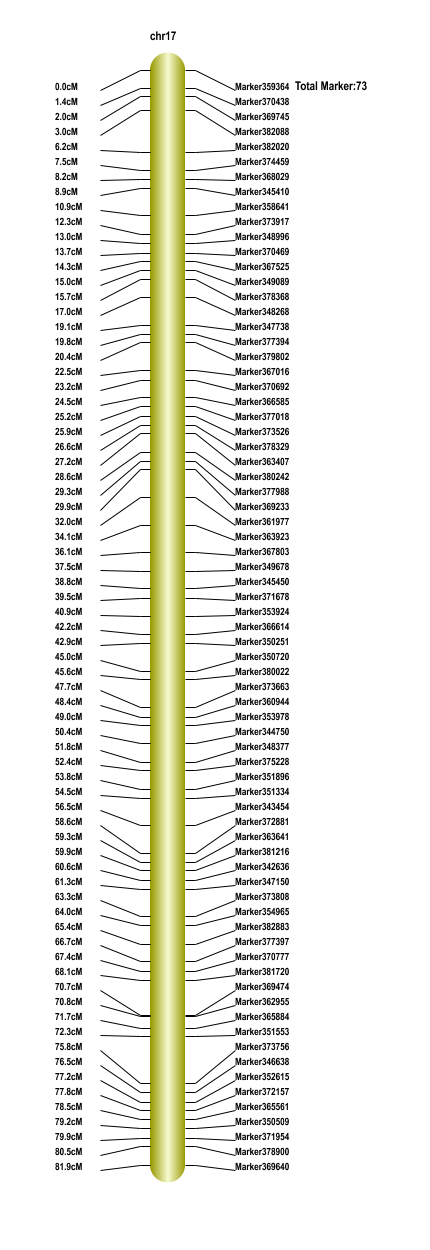

Supplement: Supplementary file 3 [file Presentation_3.ZIP › male map/Grape.chr17.png]

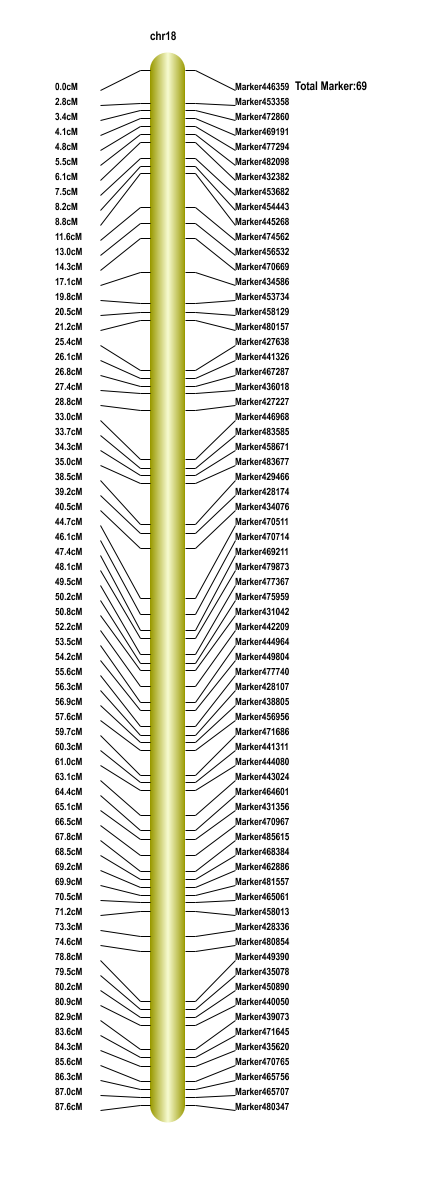

Supplement: Supplementary file 3 [file Presentation_3.ZIP › male map/Grape.chr18.png]

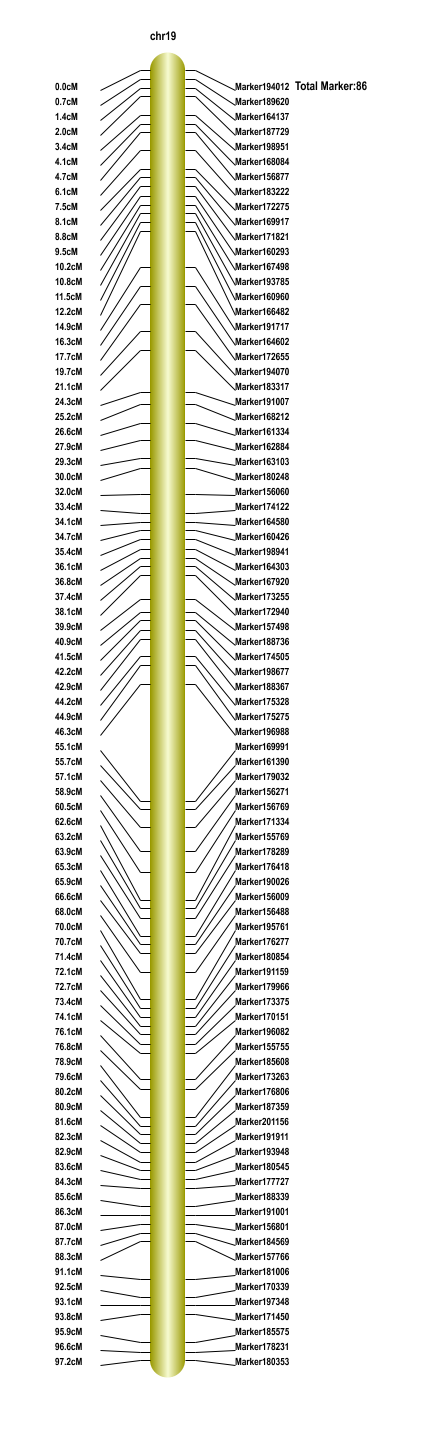

Supplement: Supplementary file 3 [file Presentation_3.ZIP › male map/Grape.chr19.png]

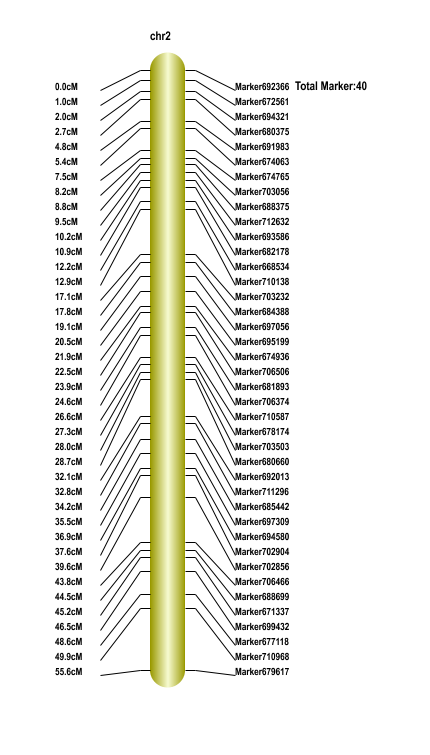

Supplement: Supplementary file 3 [file Presentation_3.ZIP › male map/Grape.chr2.png]

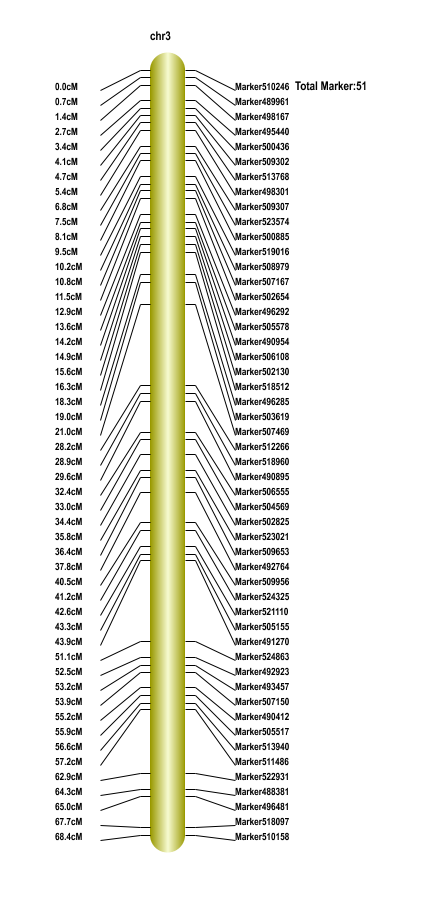

Supplement: Supplementary file 3 [file Presentation_3.ZIP › male map/Grape.chr3.png]

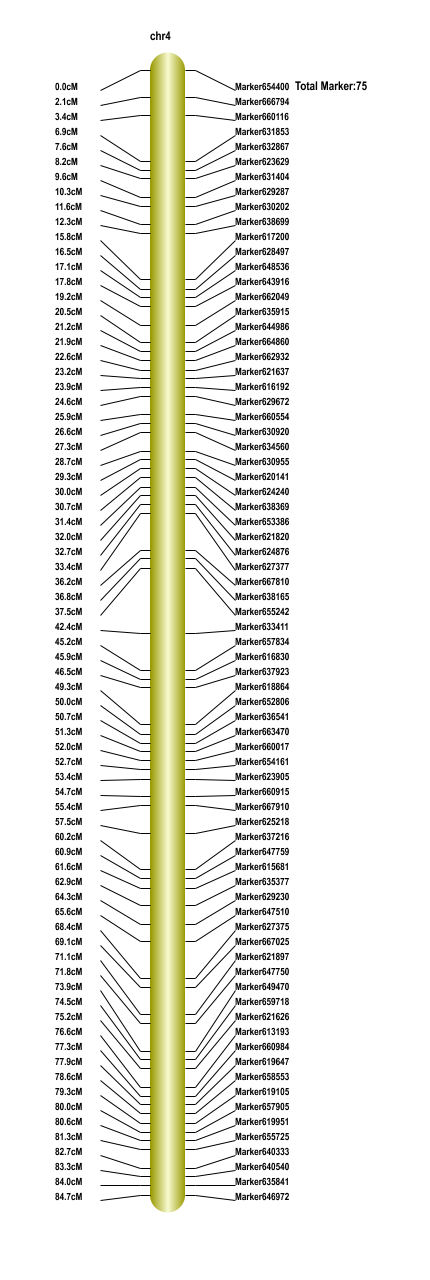

Supplement: Supplementary file 3 [file Presentation_3.ZIP › male map/Grape.chr4.png]

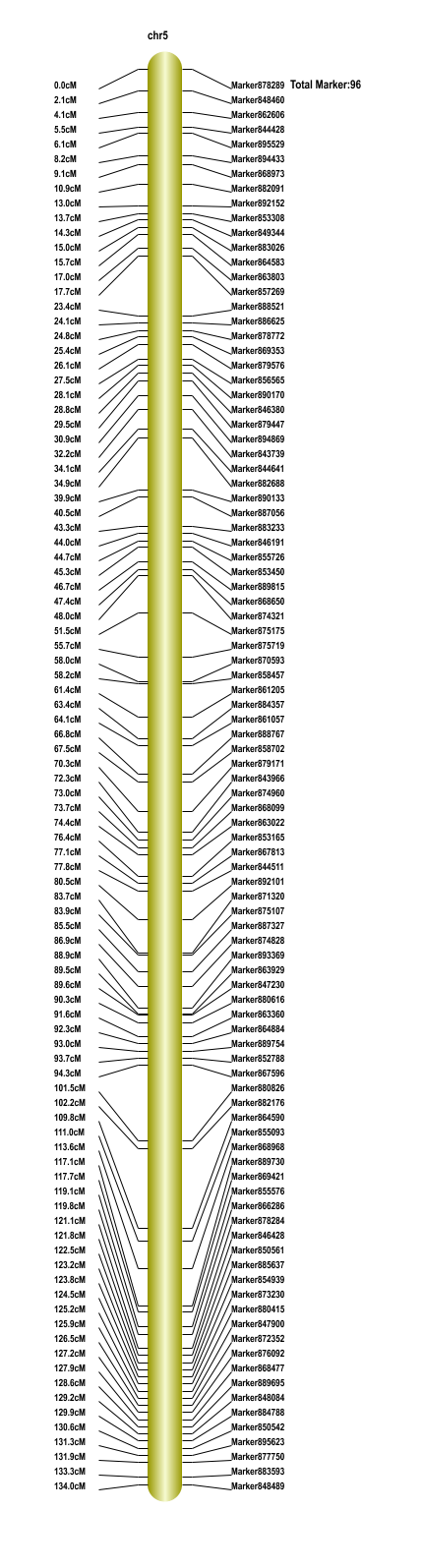

Supplement: Supplementary file 3 [file Presentation_3.ZIP › male map/Grape.chr5.png]

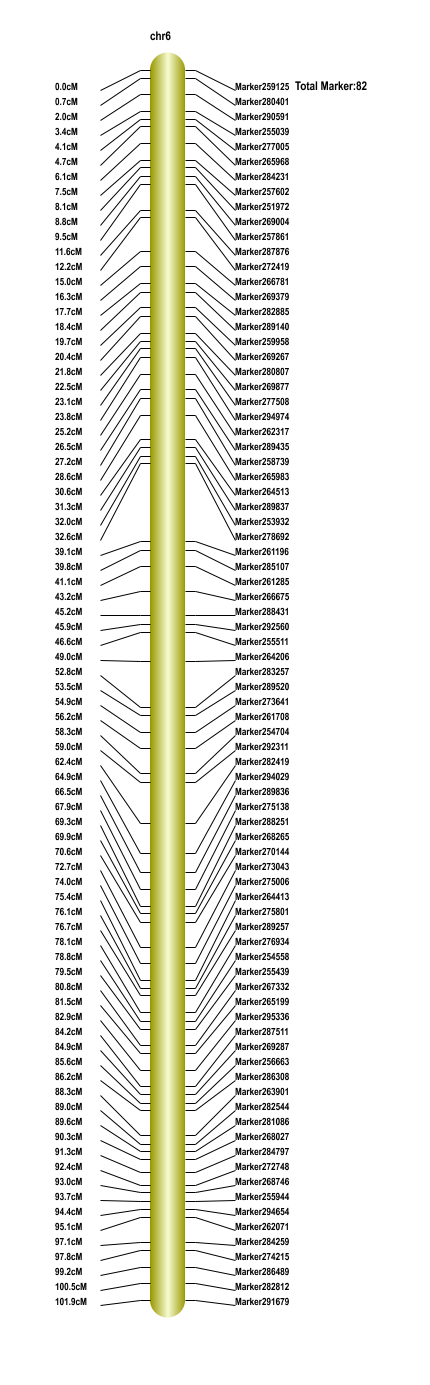

Supplement: Supplementary file 3 [file Presentation_3.ZIP › male map/Grape.chr6.png]

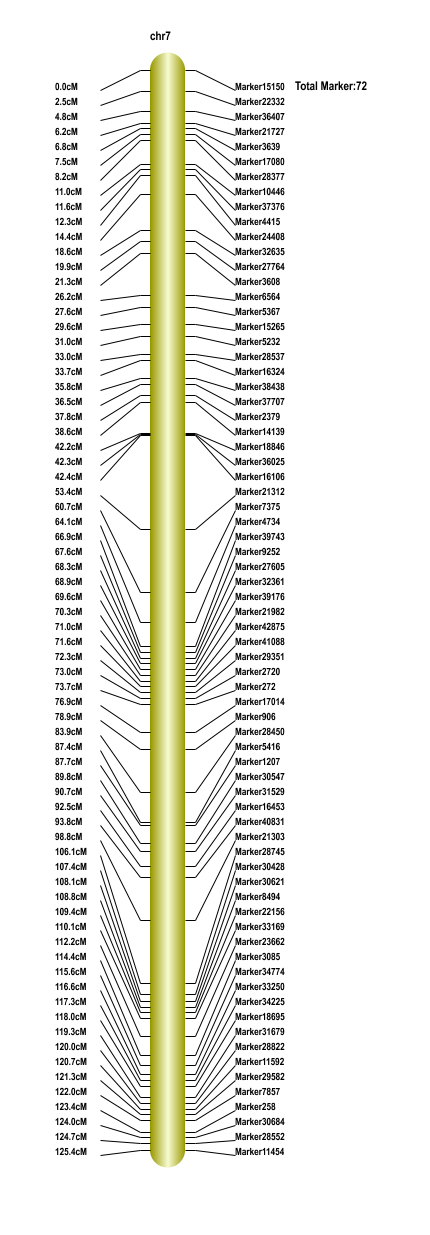

Supplement: Supplementary file 3 [file Presentation_3.ZIP › male map/Grape.chr7.png]

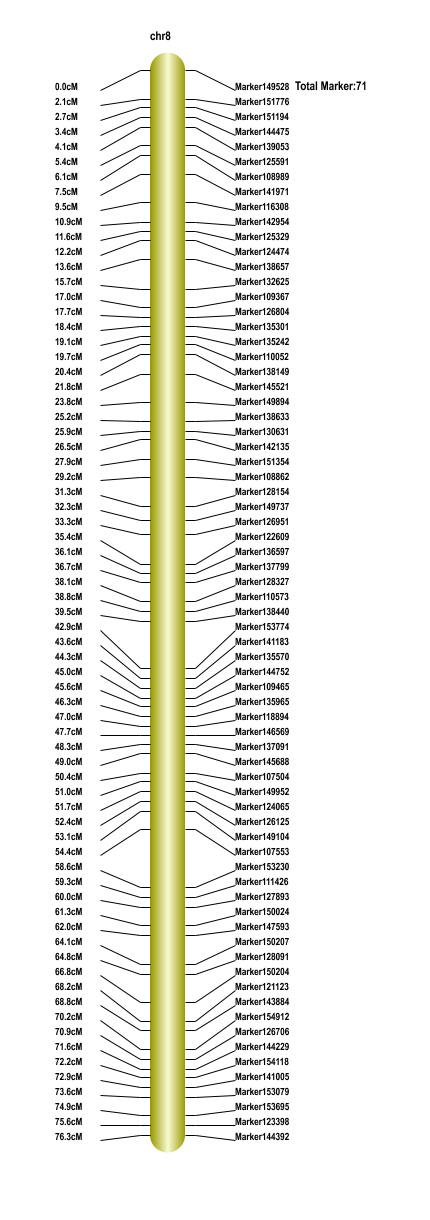

Supplement: Supplementary file 3 [file Presentation_3.ZIP › male map/Grape.chr8.png]

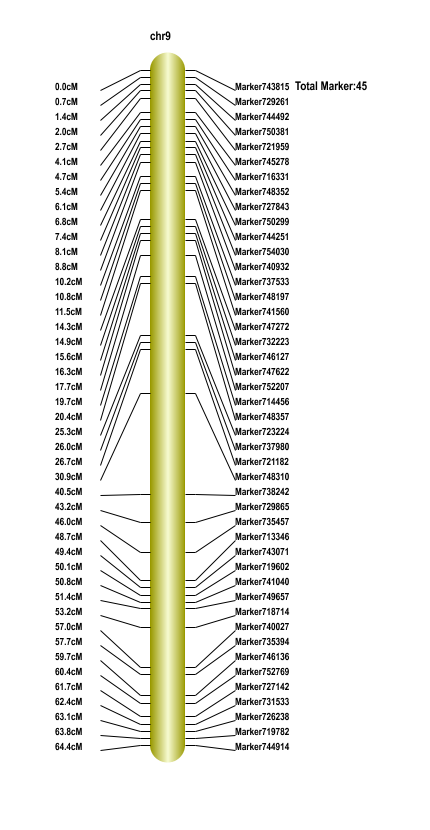

Supplement: Supplementary file 3 [file Presentation_3.ZIP › male map/Grape.chr9.png]
